# Supplementary material for: Unveiling the Bioactive Potential of the Invasive Jellyfish Phyllorhiza punctata Through Integrative Transcriptomic and Proteomic Analyses
Source: Biomolecules. 2025 Aug 4;15(8):1121. doi: 10.3390/biom15081121 (PMC12383608; doi:10.3390/biom15081121)
Supplement: Supplementary file 1 [file biomolecules-15-01121-s001.zip › Suplementary Material/FileS3/PEPFOLD4/XP_065060176_7627/clusters.html]

 PEP-FOLD clusters


Download archive of all models  here   

|  |  |  |
| --- | --- | --- |
| # | file | sOPEP |
| # cluster 1 - Size: 52 |  |  |
|  | XP\_065060176\_7627-model1 | -12.8071 |
|  | XP\_065060176\_7627-model1.1 | -12.8071 |
|  | XP\_065060176\_7627-model1.2 | -12.8071 |
|  | XP\_065060176\_7627-model1.3 | -12.7294 |
|  | XP\_065060176\_7627-model1.4 | -12.7294 |
|  | XP\_065060176\_7627-model1.5 | -12.7294 |
|  | XP\_065060176\_7627-model1.6 | -12.7294 |
|  | XP\_065060176\_7627-model1.7 | -12.7294 |
|  | XP\_065060176\_7627-model1.8 | -12.7294 |
|  | XP\_065060176\_7627-model1.9 | -12.7294 |
|  | XP\_065060176\_7627-model1.10 | -12.7294 |
|  | XP\_065060176\_7627-model1.11 | -12.7294 |
|  | XP\_065060176\_7627-model1.12 | -12.7294 |
|  | XP\_065060176\_7627-model1.13 | -12.7294 |
|  | XP\_065060176\_7627-model1.14 | -12.7294 |
|  | XP\_065060176\_7627-model1.15 | -12.7294 |
|  | XP\_065060176\_7627-model1.16 | -12.7294 |
|  | XP\_065060176\_7627-model1.17 | -12.7294 |
|  | XP\_065060176\_7627-model1.18 | -12.7294 |
|  | XP\_065060176\_7627-model1.19 | -12.7294 |
|  | XP\_065060176\_7627-model1.20 | -12.7294 |
|  | XP\_065060176\_7627-model1.21 | -12.7294 |
|  | XP\_065060176\_7627-model1.22 | -12.7294 |
|  | XP\_065060176\_7627-model1.23 | -12.7294 |
|  | XP\_065060176\_7627-model1.24 | -12.7294 |
|  | XP\_065060176\_7627-model1.25 | -12.7294 |
|  | XP\_065060176\_7627-model1.26 | -12.7294 |
|  | XP\_065060176\_7627-model1.27 | -12.7294 |
|  | XP\_065060176\_7627-model1.28 | -12.7294 |
|  | XP\_065060176\_7627-model1.29 | -12.7294 |
|  | XP\_065060176\_7627-model1.30 | -12.7294 |
|  | XP\_065060176\_7627-model1.31 | -12.7294 |
|  | XP\_065060176\_7627-model1.32 | -12.7294 |
|  | XP\_065060176\_7627-model1.33 | -12.7294 |
|  | XP\_065060176\_7627-model1.34 | -12.7294 |
|  | XP\_065060176\_7627-model1.35 | -12.7294 |
|  | XP\_065060176\_7627-model1.36 | -12.7294 |
|  | XP\_065060176\_7627-model1.37 | -12.7294 |
|  | XP\_065060176\_7627-model1.38 | -12.7294 |
|  | XP\_065060176\_7627-model1.39 | -12.7294 |
|  | XP\_065060176\_7627-model1.40 | -12.7294 |
|  | XP\_065060176\_7627-model1.41 | -12.7294 |
|  | XP\_065060176\_7627-model1.42 | -12.7294 |
|  | XP\_065060176\_7627-model1.43 | -12.7294 |
|  | XP\_065060176\_7627-model1.44 | -12.7294 |
|  | XP\_065060176\_7627-model1.45 | -12.7294 |
|  | XP\_065060176\_7627-model1.46 | -12.7294 |
|  | XP\_065060176\_7627-model1.47 | -12.7294 |
|  | XP\_065060176\_7627-model1.48 | -12.7294 |
|  | XP\_065060176\_7627-model1.49 | -12.6387 |
|  | XP\_065060176\_7627-model1.50 | -12.6387 |
|  | XP\_065060176\_7627-model1.51 | -12.6387 |
| # cluster 2 - Size: 24 |  |  |
|  | XP\_065060176\_7627-model2 | -12.7749 |
|  | XP\_065060176\_7627-model2.1 | -12.7135 |
|  | XP\_065060176\_7627-model2.2 | -12.7135 |
|  | XP\_065060176\_7627-model2.3 | -12.7135 |
|  | XP\_065060176\_7627-model2.4 | -12.7135 |
|  | XP\_065060176\_7627-model2.5 | -12.7135 |
|  | XP\_065060176\_7627-model2.6 | -12.7135 |
|  | XP\_065060176\_7627-model2.7 | -12.7135 |
|  | XP\_065060176\_7627-model2.8 | -12.7135 |
|  | XP\_065060176\_7627-model2.9 | -12.7135 |
|  | XP\_065060176\_7627-model2.10 | -12.7135 |
|  | XP\_065060176\_7627-model2.11 | -12.7135 |
|  | XP\_065060176\_7627-model2.12 | -12.7135 |
|  | XP\_065060176\_7627-model2.13 | -12.7135 |
|  | XP\_065060176\_7627-model2.14 | -12.7135 |
|  | XP\_065060176\_7627-model2.15 | -12.7135 |
|  | XP\_065060176\_7627-model2.16 | -12.7135 |
|  | XP\_065060176\_7627-model2.17 | -12.7135 |
|  | XP\_065060176\_7627-model2.18 | -12.7135 |
|  | XP\_065060176\_7627-model2.19 | -12.7135 |
|  | XP\_065060176\_7627-model2.20 | -12.7135 |
|  | XP\_065060176\_7627-model2.21 | -12.7135 |
|  | XP\_065060176\_7627-model2.22 | -12.7135 |
|  | XP\_065060176\_7627-model2.23 | -12.7135 |
| # cluster 3 - Size: 20 |  |  |
|  | XP\_065060176\_7627-model3 | -12.6816 |
|  | XP\_065060176\_7627-model3.1 | -12.6816 |
|  | XP\_065060176\_7627-model3.2 | -12.6816 |
|  | XP\_065060176\_7627-model3.3 | -12.6816 |
|  | XP\_065060176\_7627-model3.4 | -12.6816 |
|  | XP\_065060176\_7627-model3.5 | -12.6816 |
|  | XP\_065060176\_7627-model3.6 | -12.6683 |
|  | XP\_065060176\_7627-model3.7 | -12.6683 |
|  | XP\_065060176\_7627-model3.8 | -12.6683 |
|  | XP\_065060176\_7627-model3.9 | -12.6683 |
|  | XP\_065060176\_7627-model3.10 | -12.6683 |
|  | XP\_065060176\_7627-model3.11 | -12.6358 |
|  | XP\_065060176\_7627-model3.12 | -12.6358 |
|  | XP\_065060176\_7627-model3.13 | -12.6358 |
|  | XP\_065060176\_7627-model3.14 | -12.6358 |
|  | XP\_065060176\_7627-model3.15 | -12.6358 |
|  | XP\_065060176\_7627-model3.16 | -12.6358 |
|  | XP\_065060176\_7627-model3.17 | -12.6358 |
|  | XP\_065060176\_7627-model3.18 | -12.6358 |
|  | XP\_065060176\_7627-model3.19 | -12.6358 |
| # cluster 4 - Size: 2 |  |  |
|  | XP\_065060176\_7627-model4 | -12.6626 |
|  | XP\_065060176\_7627-model4.1 | -12.6626 |
| # cluster 5 - Size: 2 |  |  |
|  | XP\_065060176\_7627-model5 | -12.6535 |
|  | XP\_065060176\_7627-model5.1 | -12.6535 |
|  |  |  |
